# Supplementary material for: Null alleles are ubiquitous at microsatellite loci in the Wedge Clam (Donax trunculus)
Source: PeerJ. 2017 Apr 18;5:e3188. doi: 10.7717/peerj.3188 (PMC5398275; doi:10.7717/peerj.3188)

Without the locus D.tru2

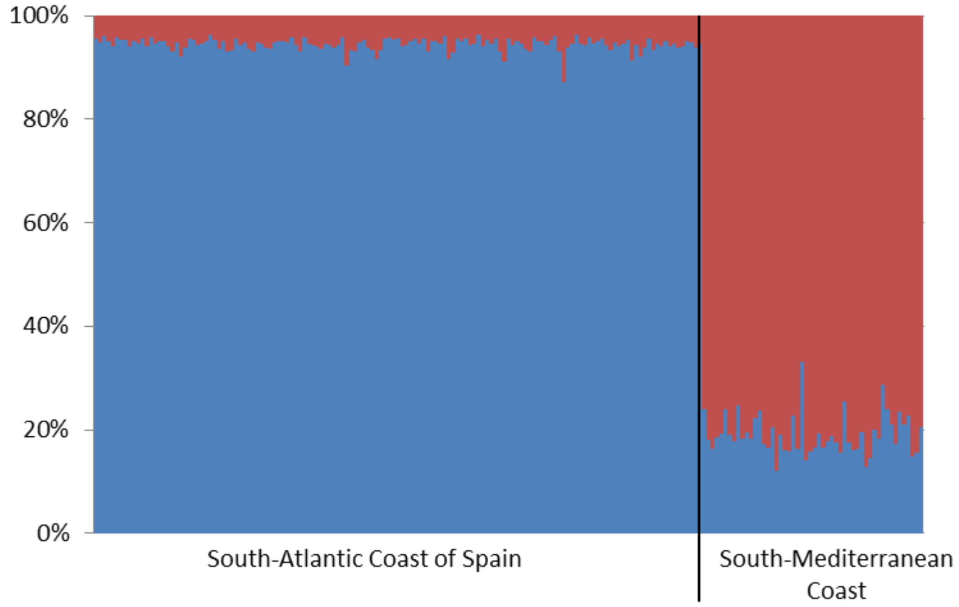

Without the locus D.tru4

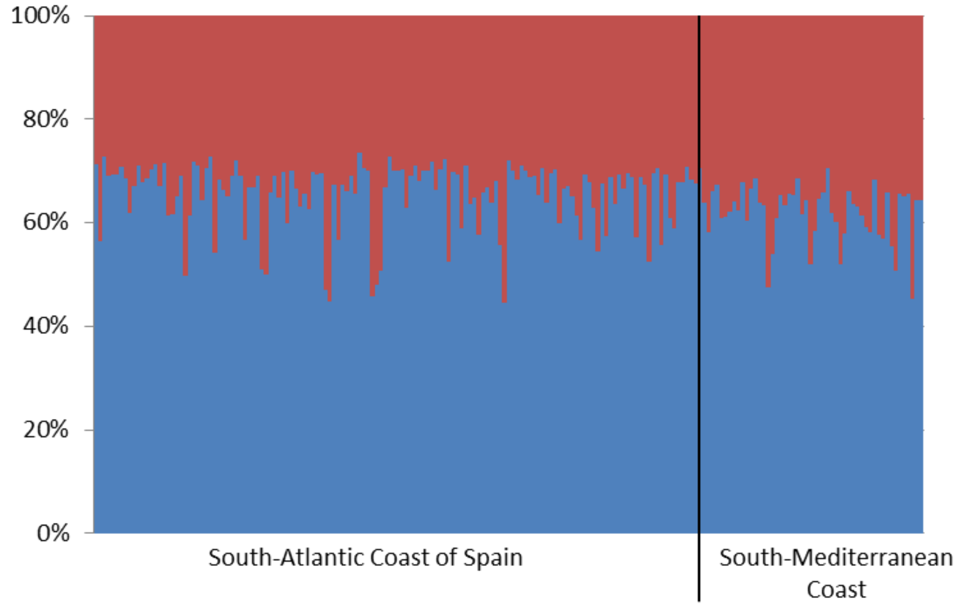

Without the locus D.tru6

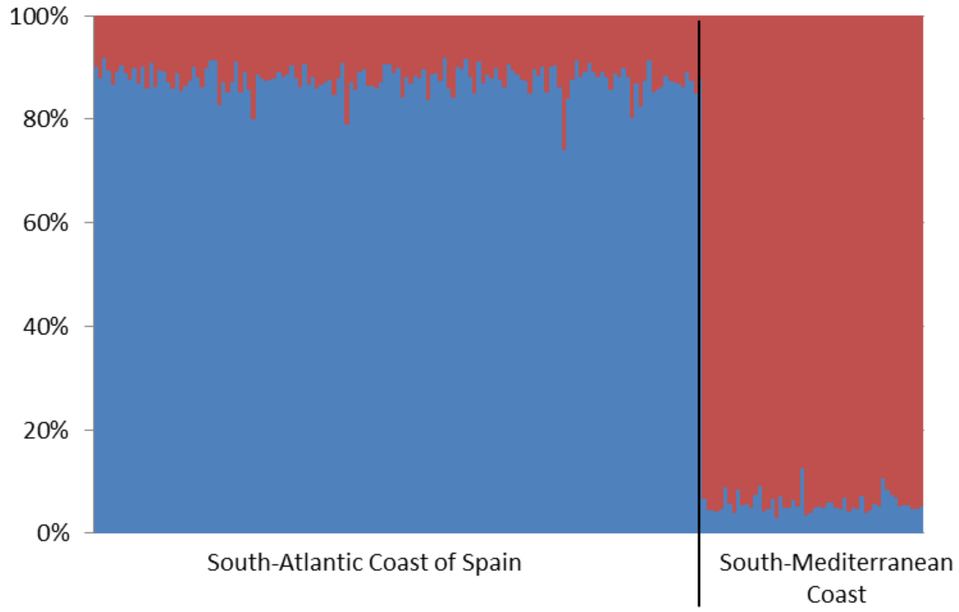

Without the locus D.tru8

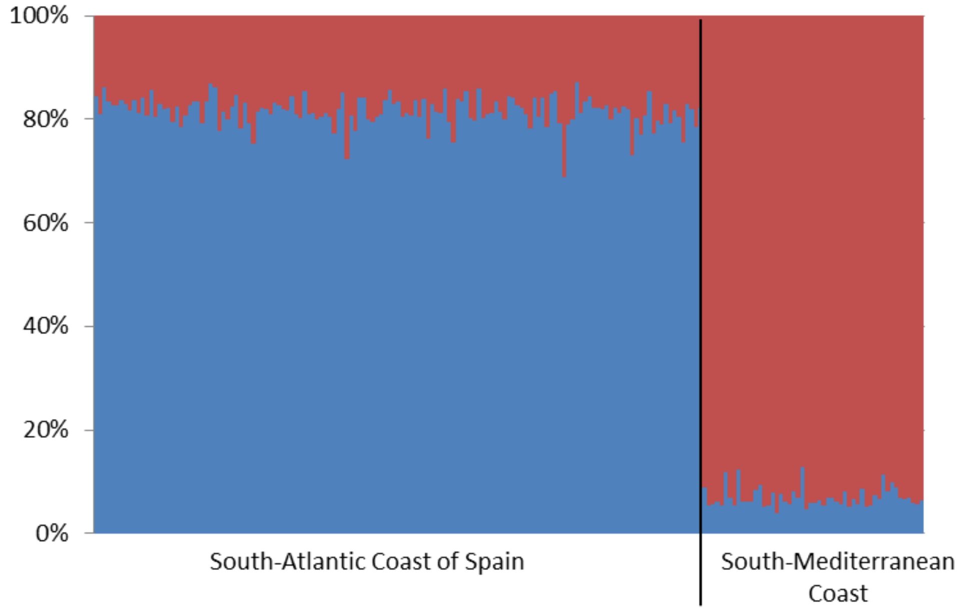

Without the locus D.tru11

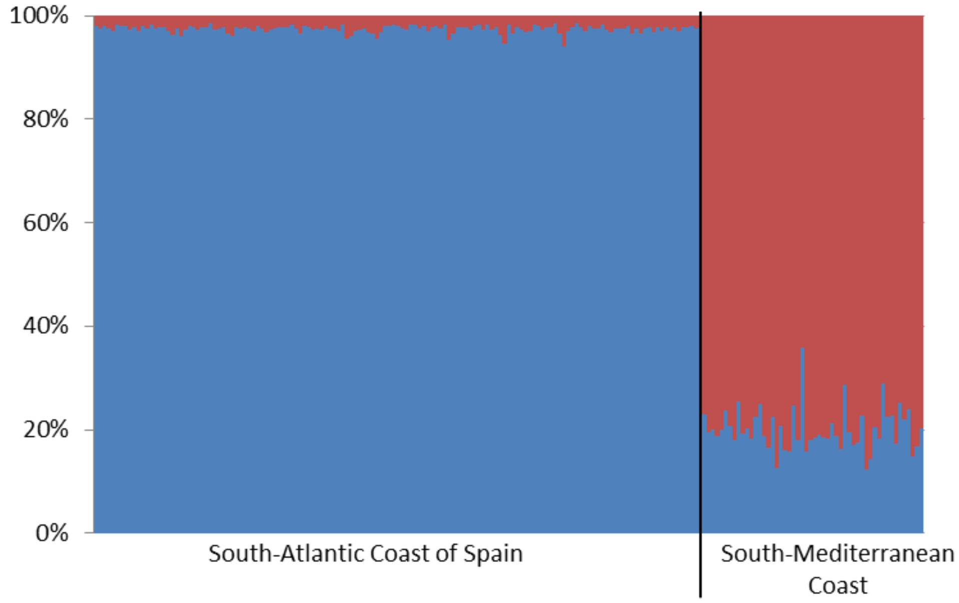

Without the locus D.tru14

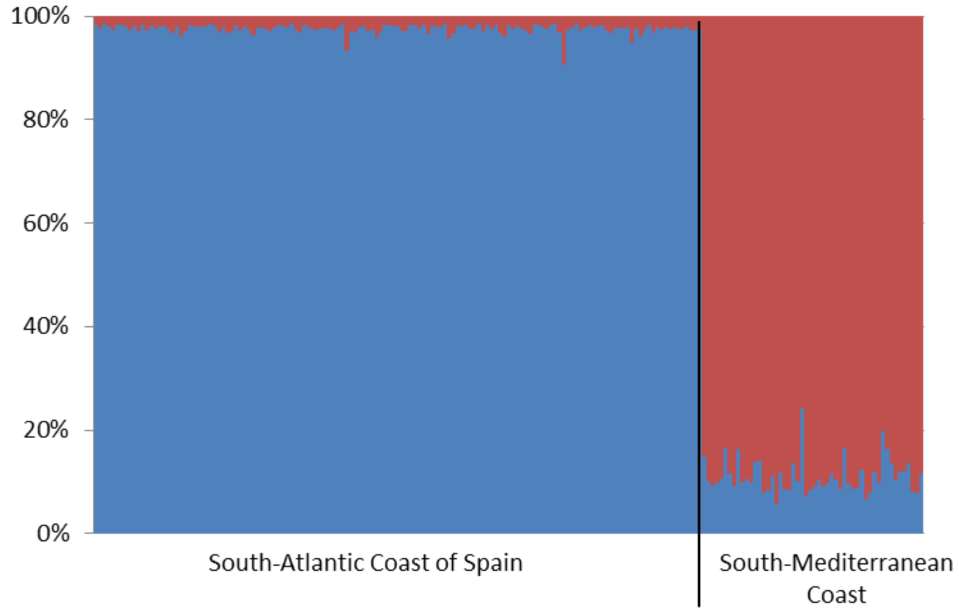

Without the locus D.tru15

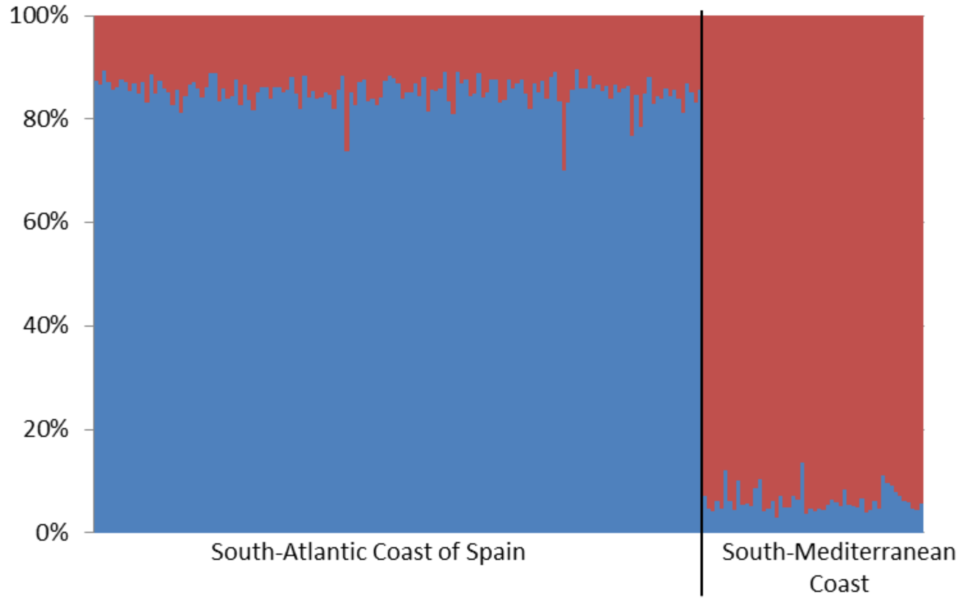

Without the locus D.tru16

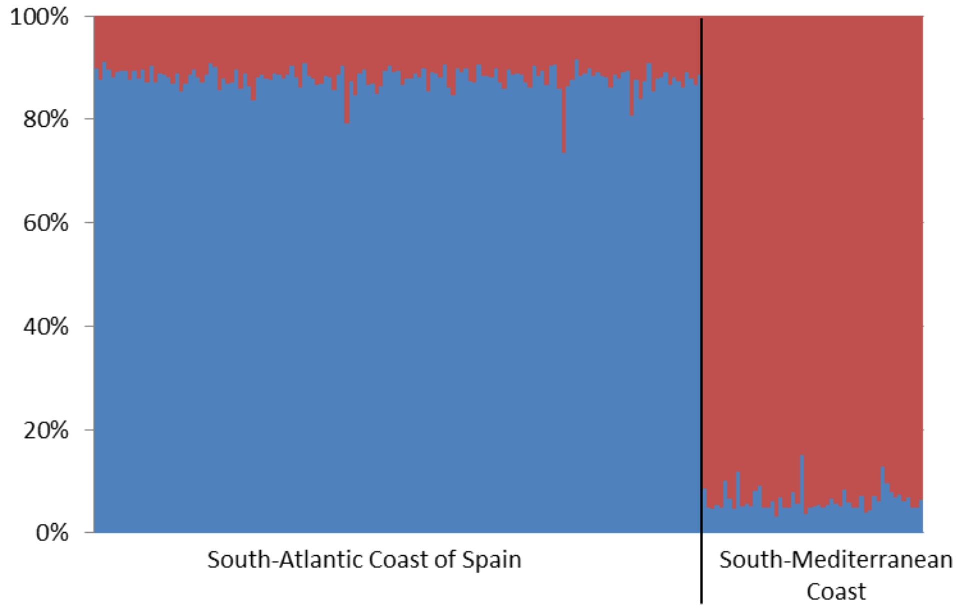

Without the locus D.tru19

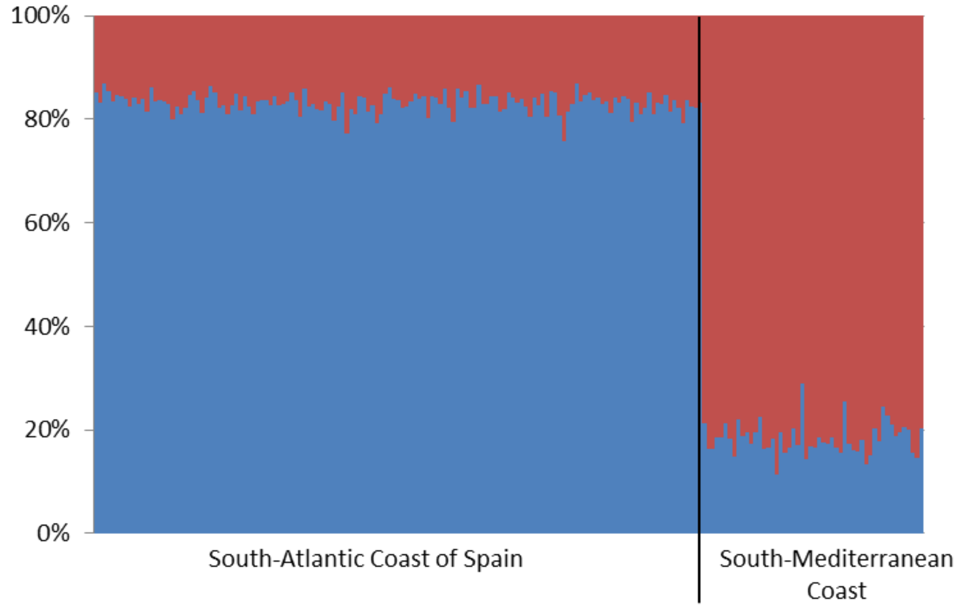

Without the locus D.tru22

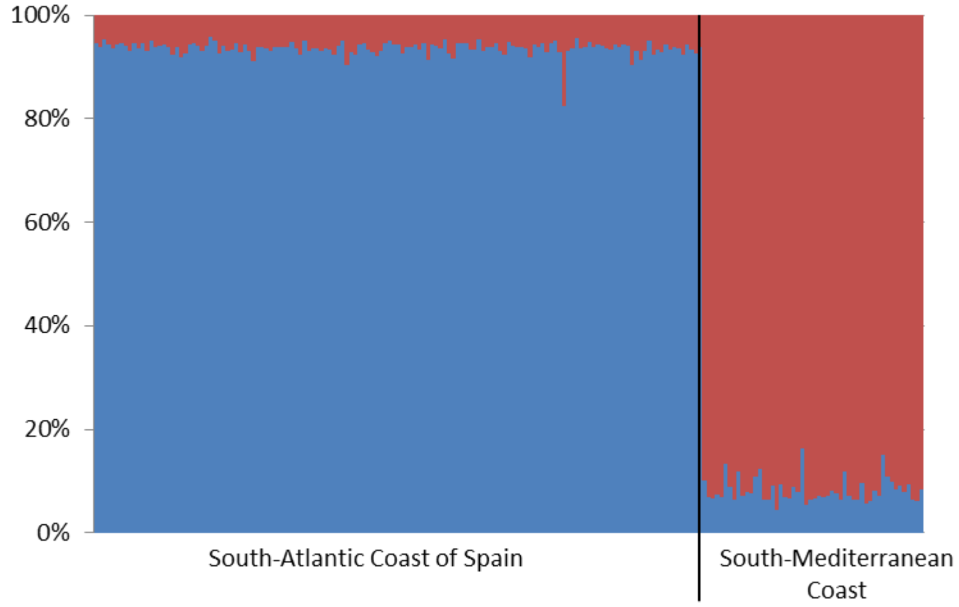

Without the locus D.tru23

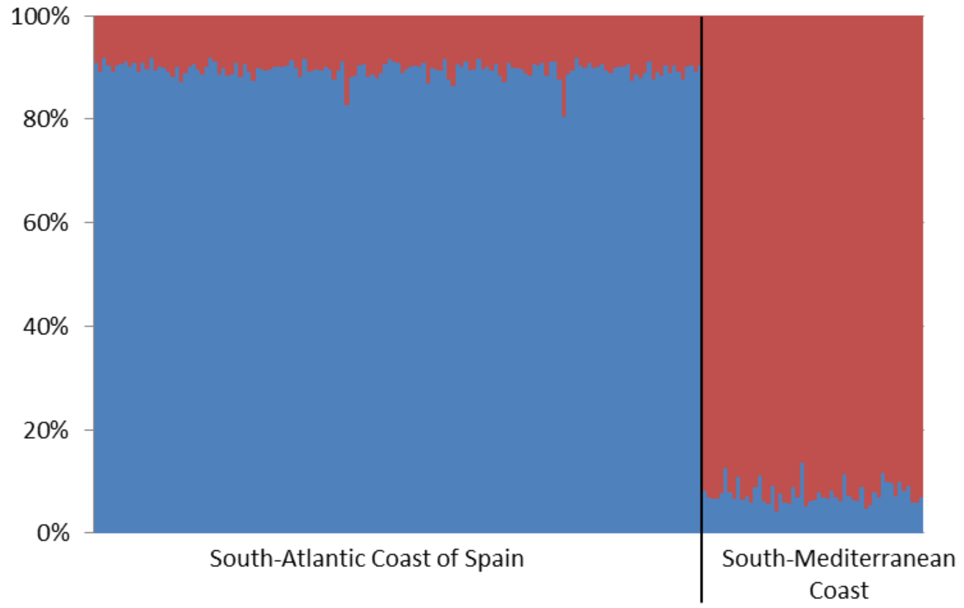

Without the locus D.tru26

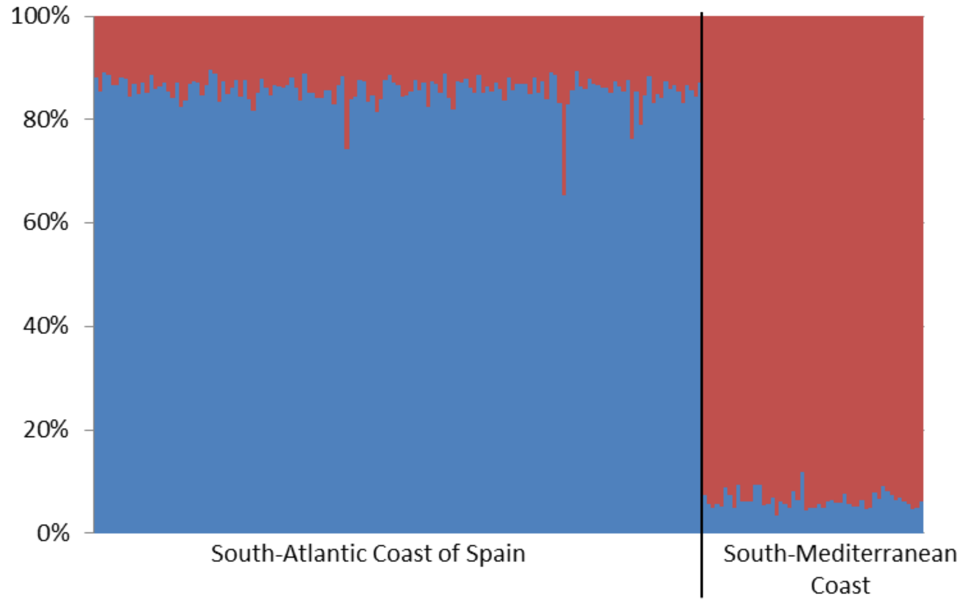

Without the locus D.tru29

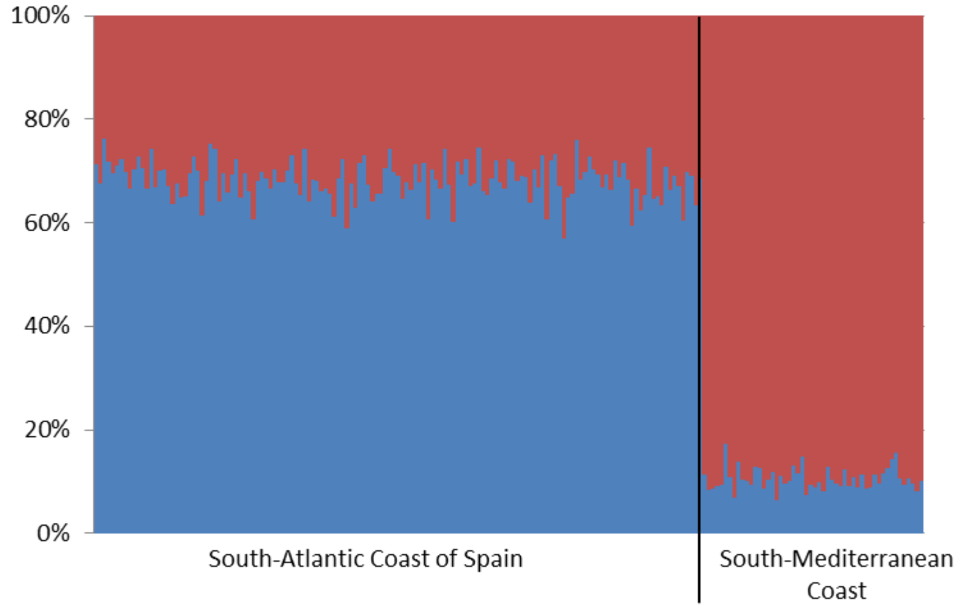

Without the locus D.tru32

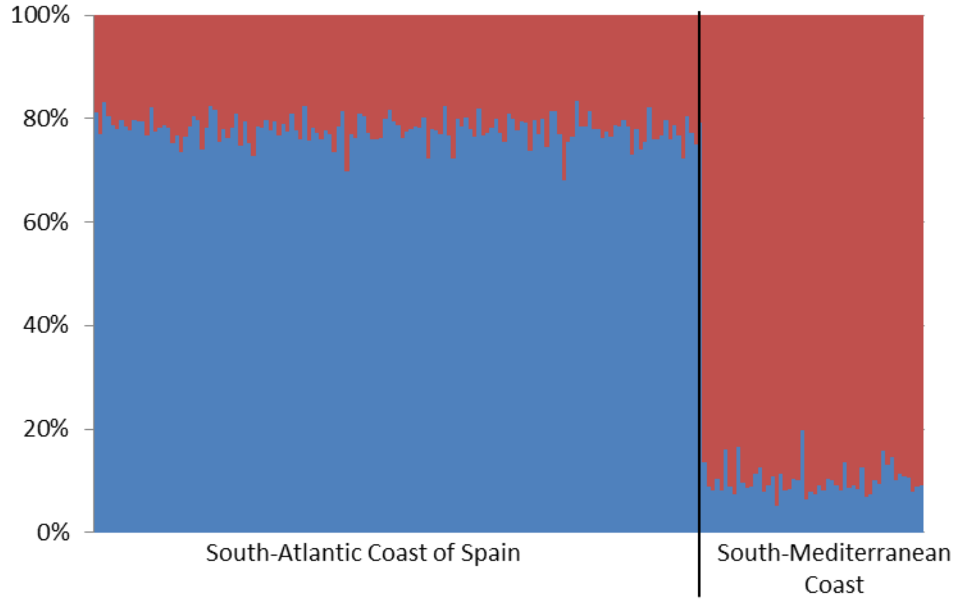

Without the locus D.tru40

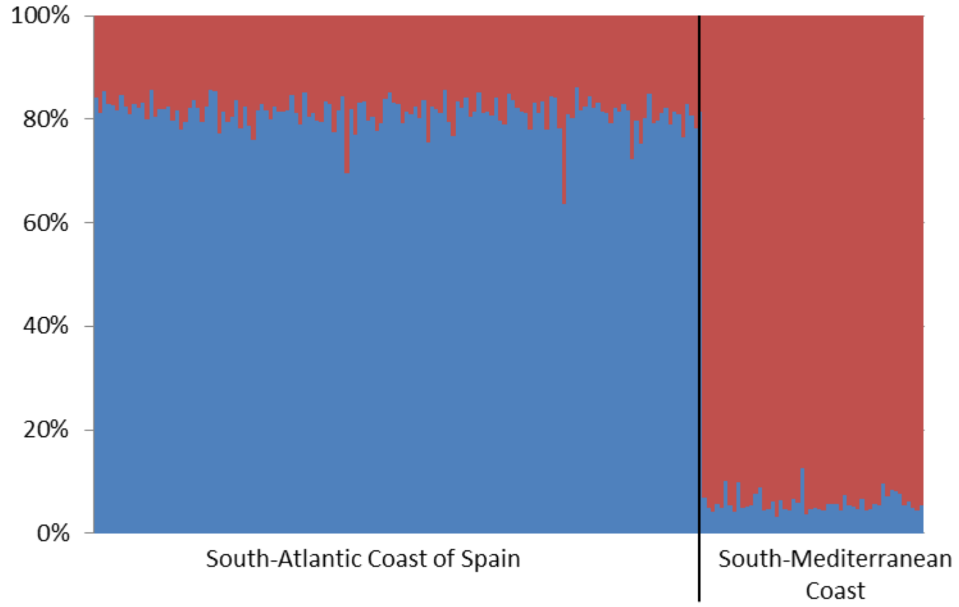

Without the locus D.tru49

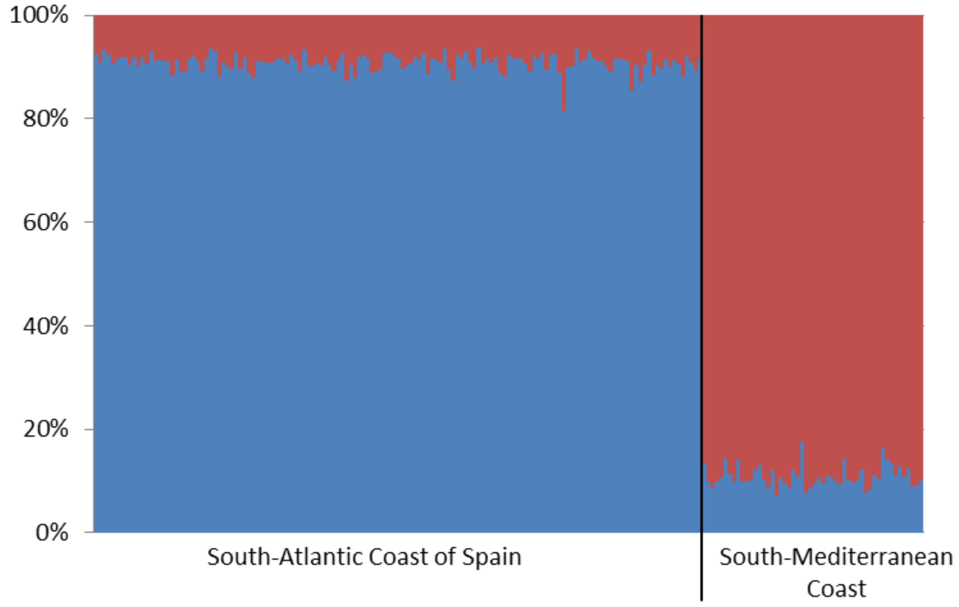

Supplement: Figure S1 — Individuals are represented as vertical bars, where the amount of each colour indicates the proportion of each inferred cluster. A represents the clusters obtained in the simulations considering the absence of null alleles while B represents those assuming them. Isla Canela and Doñana are represented by the label “South-Atlantic Coast of Spain”, and Caleta de Vélez by the label “South-Mediterranean Coast”. [file peerj-05-3188-s001.pdf]
